# Sphingolipid Distribution, Content and Gene Expression during Olive-Fruit Development and Ripening

**DOI:** 10.3389/fpls.2018.00028

**Published:** 2018-01-26

**Authors:** Carla Inês, Maria C. Parra-Lobato, Miguel A. Paredes, Juana Labrador, Mercedes Gallardo, Mariana Saucedo-García, Marina Gavilanes-Ruiz, Maria C. Gomez-Jimenez

**Affiliations:** ^1^Department of Plant Physiology, University of Extremadura, Badajoz, Spain; ^2^Department of Plant Physiology, University of Vigo, Vigo, Spain; ^3^Institute of Agricultural Sciences, Autonomous University of the State of Hidalgo, Tulancingo, Mexico; ^4^Departamento de Bioquímica, Facultad de Química, Universidad Nacional Autónoma de México, Mexico City, Mexico

**Keywords:** alkaline dihydroceramidase, fruit ripening, glucosylceramidase, olive, serine palmitoyltransferase, sphingolipid, sphingosine kinase, vesicle trafficking

## Abstract

Plant sphingolipids are involved in the building of the matrix of cell membranes and in signaling pathways of physiological processes and environmental responses. However, information regarding their role in fruit development and ripening, a plant-specific process, is unknown. The present study seeks to determine whether and, if so, how sphingolipids are involved in fleshy-fruit development and ripening in an oil-crop species such as olive (*Olea europaea* L. cv. Picual). Here, in the plasma-membranes of live protoplasts, we used fluorescence to examine various specific lipophilic stains in sphingolipid-enriched regions and investigated the composition of the sphingolipid long-chain bases (LCBs) as well as the expression patterns of sphingolipid-related genes, *OeSPT*, *OeSPHK*, *OeACER*, and *OeGlcCerase*, during olive-fruit development and ripening. The results demonstrate increased sphingolipid content and vesicle trafficking in olive-fruit protoplasts at the onset of ripening. Moreover, the concentration of LCB [t18:1(8*Z*), t18:1 (8*E*), t18:0, d18:2 (4*E*/8*Z*), d18:2 (4*E*/8*E*), d18:1(4*E*), and 1,4-anhydro-t18:1(8*E*)] increases during fruit development to reach a maximum at the onset of ripening, although these molecular species decreased during fruit ripening. On the other hand, *OeSPT*, *OeSPHK*, and *OeGlcCerase* were expressed differentially during fruit development and ripening, whereas *OeACER* gene expression was detected only at the fully ripe stage. The results provide novel data about sphingolipid distribution, content, and biosynthesis/turnover gene transcripts during fleshy-fruit ripening, indicating that all are highly regulated in a developmental manner.

## Introduction

The olive (*Olea europaea* L.) is one of the most economically important fruit trees worldwide for the oil of its fruit. During development and ripening, the olive fruit undergoes considerable physical and biochemical changes, with five distinguishable phases: (1) fruit set following fertilization, (2) seed development, (3) endocarp lignification, (4) mesocarp development, and (5) ripening (Conde et al., 2008). Fruit tissues during these phases undergo additional biochemical and physiological transformations including cell division and enlargement, oil production, metabolite build-up, mesocarp softening, phenol breakdown, and coloration change (owing to anthocyanin accumulating in the outer mesocarp) (Conde et al., 2008). Oil synthesis starts after endocarp lignification, while the phenolic fraction is maximal at fruit set and decreases rapidly over fruit development (Conde et al., 2008).

Because of its high commercial value, the lipid content of olive fruit has received special attention regarding composition and dynamics of the olive-oil components. The most extensively studied is the glycerolipid fraction of triglycerides (Bates, 2016). However, the biology and dynamics of other lipids has been left behind (Lynch and Dunn, 2004). This is true for sphingolipids, the other class of lipids that together with the glycerolipids are the most abundant lipids in plants (Sperling et al., 2005; Chen et al., 2006). Quantitatively, complex sphingolipids have been assumed to be the species most represented in plants. These are localized mainly in vacuole and plasma-membranes, but are also found in the *trans*-Golgi network and in recycling endosomes (Simons and Toomre, 2000). In these allocations, complex sphingolipids function as structural components of the membrane matrix. However, small amounts of complex sphingolipid precursors, which are transiently produced, have been involved as second messengers in a variety of physiological plant processes, such as programmed cell death (Saucedo-García et al., 2011), guard-cell closure (Worrall et al., 2008; Guo et al., 2012), cell polarity (Markham et al., 2011), and responses to stress such as low temperatures (Chen et al., 2012; Dutilleul et al., 2012) and pathogens (Peer et al., 2010).

The pathway of sphingolipid synthesis appears to be very similar in all the plant species studied (Luttgeharm et al., 2016). The first step is the generation of the long-chain base (LCB) backbone, through the condensation of serine with palmitoyl-CoA to generate 3-ketosphinganine which is reduced to yield sphinganine, a C18 dihydroxy-LCB. Subsequent modifications on sphinganine structure, such as hydroxylation and/or desaturation, can take place by specific hydroxylases or desaturases. In plants, hydroxylation occurs predominantly in C4 of sphinganine to generate phytosphingosine. The double bond between C4 and C5 occurs exclusively in the *trans* configuration and/or between C8 and C9 of LCBs, which can be either *cis* or *trans* (Chen et al., 2009). Then, sphinganine is linked by the amino group in the C2 to a fatty acid through an amide linkage, forming a ceramide. This reaction is catalyzed by a ceramide synthase (CS). Arabidopsis genome contains three *CS* genes called *LOH1-LOH3*. LOH1 and LOH3 have preference for trihydroxy-LCBs and acyl chains with 20 to 26C [very long-chain fatty acids (VLCFA)], while LOH2 is selective for dihydroxy-LCBs and C16 acyl chains (a long chain-fatty acid or LCFA). The combination of 9 different LCBs and 32 different FAs yields 288 potential ceramide backbones (Markham et al., 2013). The ceramide can bind polar heads in the C1 to form glucosylceramide (GlcCer) or a phosphoinositol moiety, to form the the major complex sphingolipids families found in plants: GlcCers and glucosylinositolphosphoceramides (GIPCs) (Chen et al., 2009). The variety of the different carbohydrate molecules added to the polar head is the other factor that introduces diversity to complex sphingolipids.

Alternatively, LCBs not used in ceramide formation can be phosphorylated by LCB kinases (LCB-P), which can be either dephosphorylated by LCB-P phosphatases or cleaved by LCB-P lyases to yield a long-chain aldehyde and ethanolamine phosphate.

Little is known about plant enzymes involved in the catabolism of sphingolipids to regulate sphingolipid homeostasis. For ceramide turnover, the eukaryotic cells use ceramidases. Ceramides are degraded to LCBs and fatty acids. According to their optimal pH preferences, ceramidases are classified as acidic, neutral, and alkaline (Mao and Obeid, 2008). In plants, no homologs of acid ceramidases have been identified, but plants possess neutral and alkaline ceramidases (Wu et al., 2015). Arabidopsis contains three predicted neutral ceramidases and one homolog of alkaline ceramidase (Wu et al., 2015). The Arabidopsis alkaline ceramidase (AtACER) functions in disease resistance against *Pseudomonas syringae* and in salt tolerance. AtACER shows no substrate preference based on the length of fatty acid moieties but according to LCB moieties, it acts preferentially on hydroxyceramides (Wu et al., 2015). The neutral ceramidase 1 in Arabidopsis has preference for hydroxyceramides containing t18:0 or t18:1 (Li et al., 2015). The degradation of complex sphingolipids such as GlcCer is catalyzed by glucosylceramidase (GlcCerase) in mammals, but no homolog has been identified in Arabidopsis. The turnover of GIPC has not yet been determined in Arabidopsis (Chen et al., 2009).

There are a few examples wherein sphingolipids are important in the plant development. Experimental work has clearly established that sphingolipids are essential to sustain the vegetative growth of Arabidopsis (Dietrich et al., 2008) but also for adequate pollen development and competence (Chen et al., 2008; Rennie et al., 2014). Recently, we reported the first evidence for the potential involvement of sphingolipids in abscission processes as well as an increase in the relative content of sphingolipids during mature-fruit abscission in live protoplasts from abscission zone cells (Gil-Amado and Gomez-Jimenez, 2013; Parra-Lobato et al., 2017). However, information regarding their possible involvement in fleshy-fruit development and ripening, a plant-specific process, is still lacking.

The purpose of this study was to investigate the developmental regulation of the sphingolipid biosynthesis and turnover pathways during fruit development and ripening in olive. Although the results of lipid analyses during olive-fruit development from several cultivars have been available for many years (D’Angeli and Altamura, 2016), a characterization of the sphingolipid content is still lacking. For this purpose, the visualization of the sphingolipid enriched regions in the plasma-membranes of live protoplasts from olive fruit together with the LCB composition of sphingolipids, as well as the expression patterns of sphingolipid-related genes during olive-fruit development and ripening are reported. In particular, we explore the transcriptional regulation of olive genes encoding the serine palmitoyltransferase I (*OeSPT*), sphingosine kinase (*OeSPHK*), alkaline dihydroceramidase (*OeACER*), and glucosylceramidase (*OeGlcCerase*), during fruit development and ripening. The results demonstrate that endogenous sphingolipid levels are intricately controlled during fruit development, mainly involving the sphingolipids containing the LCBs t18:1(8*Z*), t18:1 (8*E*), t18:0, d18:2 (4*E*/8*Z*), d18:2 (4*E*/8*E*), d18:1(4*E*), and 1,4-anhydro-t18:1(8*E*). This detailed temporal profile of the LCB composition and the sphingolipid metabolic gene expression in the olive fruit offers new insights for understanding the physiological role(s) of plant sphingolipids. This is the first report available describing the regulation of sphingolipid distribution, content, and metabolic gene expression during fleshy-fruit development and ripening.

## Materials and Methods

### Plant Material

**FIGURE 1:**
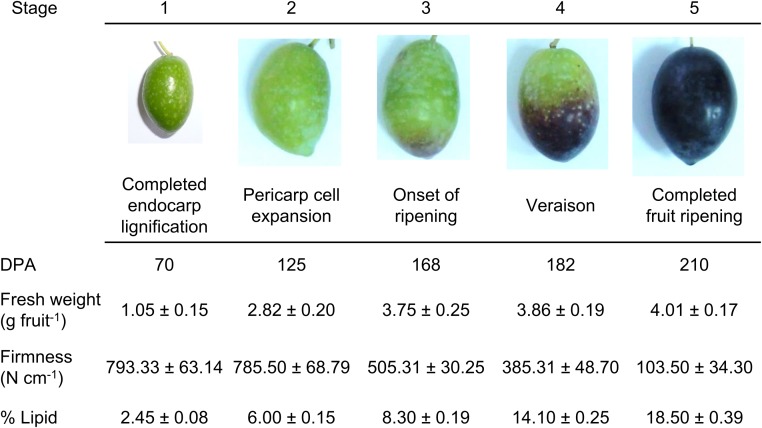
Developmental stages of the olive fruit of the cv. Picual. Stage 1: ‘completed pit hardening’; Stage 2: ‘peak of cell expansion’; Stage 3: ‘the onset of ripening’; Stage 4: ‘veraison’; Stage 5: ‘fully ripe.’ DPA: days post-anthesis. Fresh weight and firmness measurements were performed on whole fruits. Lipid content measurements were performed on fruit pericarps. Asterisks indicate statistically significant changes (*P* < 0.05) with respect to the preceding point. Data are the means of three independent experiments ± SE.

Twenty-year-old olive trees (*O. europaea* L. cv. Picual) grown under drip irrigation and fertirrigation during the 2013–2014 growing seasons in an orchard near Badajoz (Spain) were studied. To investigate fruit development, five different stages were differentiated attending to macroscopic differences. Harvested whole fruits were sampled at 70 (stage 1), 125 (stage 2), 168 (stage 3), 182 (stage 4), and 210 (stage 5) days post-anthesis (DPA) (**Figure 1**). A total of 250 fruits from 10 olive trees were used for each developmental stage. In order to minimize the effects related to asynchronous fruits ripening within the same tree, fruits with similar pigmentation were picked from all around the external parts of the tree canopy. Fruit firmness was measured at four equatorial regions of the fruit using a penetrometer (model SMT-T-50, Toyo Baldwin, Tokyo, Japan) fitted with an 5 mm plunger. For protoplast isolation, an initial group of pericarp (fruit mesocarp and epicarp) samples was used from different developmental stages, while a second group of pericarp (fruit mesocarp and epicarp) samples was frozen immediately using liquid nitrogen and then stored at -80°C for LCB analysis after sphingolipid hydrolysis, total lipids, and RNA extraction.

### Lipid Extraction

Total lipids were extracted from 800 mg samples of freeze-dried powder following Laffargue et al. (2007) procedure. Extracted lipids for each stage of development from three different extractions were quantified gravimetrically after evaporation until dryness under nitrogen at 40°C.

### Olive-Fruit Protoplast Isolation

The olive-fruit pericarps at the five different developmental stages were diced for incubation (2 h at 28°C) in a digestion buffer using a shaker at 30 rpm. The digestion buffer was composed of pectolyase (0.1% w/v), cellulase (0.8% w/v), bovine serum albumin (0.5% w/v), and polyvinylpyrolidone (0.5% w/v), all from Sigma–Aldrich, Spain. Lastly, calcium chloride (1 mM) was added. Also, the pH was held to 5.6 (MES-Tris), and, using sorbitol, the osmolarity was adjusted to 280 milliosmol/kg. Following incubation, a 10-min centrifugation was needed (80–100 rpm at 4°C) for the separation of protoplasts from pericarp fragments.

### Dye Staining of Protoplasts

Protoplast images were taken by a FluoView 1000 spectral confocal microscope (Olympus, Tokyo, Japan), which had 405, 488, 543, and 633 nm laser lines. The selection of fluorescence acquisition was made to maximize the fluorochrome emission in each case by the use of spectral detectors. Each sample comprised about 20 protoplasts, the data being compiled by FV10 4.2 software (Olympus).

### Bodipy 505/515

For the detection of neutral (apolar) lipids (Thermofisher), bodipy (4,4-difluoro-3a,4adiaza-s-indacene), a strongly lipophilic neutral fluorophore, was used. Bodipy is used for labeling a broad range of lipids, including phospholipids, fatty acids, ceramides, cholesterol, and cholesteryl esters (Elle et al., 2010). For 30 min protoplasts were incubated in darkness in protoplast buffer, their final concentration being 50 ng/ml. The spectral properties were 515 nm of emission wavelength and 505 nm excitation wavelength.

### Nile Red

Nile Red (9-(Diethylamino)-5H benzo [α] phenoxazin-5one) staining was used to detect neutral and polar lipids. This compound is a fluorescent lipophilic dye with an emission shift from red to yellow according to the degree of hydrophobicity of lipids. Nile Red is an ideal probe for detecting lipids as it exhibits high affinity, specificity, and sensitivity to the degree of hydrophobicity of lipids (Diaz et al., 2008). When this dye is excited at 515 nm, it shows three emission bands centered at 530, 580, and 636 nm corresponding to non-polar, total, and polar lipids, respectively. In the present study, the Nile Red solution was added (0.1 mg/ml in acetone) to the protoplasts, which were incubated for 10 min. Following a single washing in protoplast buffer, confocal laser scanning microscopy was used to examine the stained protoplasts. With a 585-nm long-pass filter the Nile Red orange/red fluorescence was detected, and the mean intensities of the fluorescence of the olive-fruit protoplasts were measured at the five different developmental stages.

### Bodipy-Sphingomyelin FL C12 (BD-SM)

The amphiphilic BD-SM lipid analog (*N*-(4,4-difluoro-5,7-dimethyl-4-bora-3a,4a-diaza-s-indacene-3-dodecanoyl) sphingosyl phosphocholine) is composed of a sphingosine moiety linked by an amide bond to a fatty acid. The sphingomyelin itself is covalently linked to a Bodipy fluorophor (Thermofisher). Although sphingomyelin is not present as such in plants, the structure has the features of a complex sphingolipid. Plants have an enormous variety of these lipids and they show a physicochemical behavior expected for a complex sphingolipid, therefore, BD-SM molecules incorporate into the plasma-membrane and mixed with native sphingolipids (Blachutzik et al., 2012; Parra-Lobato et al., 2017). Referring to information of the manufacturer, BD-SM features the same stereochemical conformation as biologically active sphingomyelins, despite the fluorescence labeling. As mentioned above, Bodipy’s spectral properties are 505 nm of excitation wavelength and 515 nm of emission wavelength. DMSO was used to solve the dye to a stock concentration of 1.0 μg/μl. Next, protoplasts were stained at 1% (v/v) of stock solution in protoplast buffer for 20 min at room temperature.

### FM4-64

The lipophilic FM4-64 dye (*N*-(3-triethylammoniumpropyl)-4-(6-(4-(diethylamino) phenyl)hexatrienyl)pyridiumdibromide) is based on a polyethylene structure. FM4-64 is employed extensively for studying vesicle organelle organization, trafficking, and endocytosis in eukaryotic cells (Bolte et al., 2004). According to the manufacturer (Thermofisher), the dye, water soluble, is non-toxic for living cells; cell viability was corroborated by Trypan blue application, after solving the dye in purified water to a stock solution of 1 μg/μl. After several experiments to optimize staining, a final concentration of 0.5% (v/v) and an incubation period of 10 to 15 min at room temperature in protoplast buffer were selected to stain the olive protoplasts. A wavelength of 543 nm was employed to excite the fluorophore; the emission spectrum of FM4-64 was in the range of 580–650 nm (emission maximum 640 nm).

### Cell Viability

As a cell-viability indicator, Trypan blue stain was used at 0.4% (Sigma–Aldrich, Spain). The dye was solved in 0.6 M mannitol to 1 mg/ml. Cells that are non-viable take up the dye, appearing blue, whereas intact cells and membranes resist staining. After the dye was applied directly to the protoplast buffer, the suspension was thoroughly mixed. For sharp black-and-white contrast needed for imaging, Trypan blue was used at concentrations reaching 20% (v/v). Incubation for 10 min at room temperature sufficiently stained the cells (**Supplementary Figure S1**).

### Sphingolipid Analysis

As previously described, total sphingolipids were analyzed using their released LCB (Markham et al., 2006). The samples (fruit mesocarp and epicarp) were standardized by adding C20-4-SPH (d20:1) as the internal standard. The hydrolysis of the sphingolipids followed Morrison and Hay (1970), modified after Cahoon and Lynch (1991), and *o*-phthaldialdehyde was used for derivatization. Agilent Technologies HPLC Infinity 1260 were used for the HPLC analyses using reverse-phase HPLC on a 2.1 mm × 150 mm Eclipse XBD-C18 Narrow-bore column (Agilent Technologies, Inc., Palo Alto, CA, United States). The samples were eluted (at 0.4 ml/min) with 20% RA solvent (5 mM potassium phosphate, pH 7.0), 80% solvent RB (100% methanol) for 7 min, rising to 90% solvent RB at 15 min, after which isocratic flow was applied for 10 min prior to increasing to 100% solvent RB at 30 min and a 3-min 100% solvent RB wash preceding a return to 80% solvent RB and 2 min of re-equilibration. At 340 nm, fluorescence was excited and then detected at 455 nm. The results were both analyzed as well as integrated by Openlab.

### RNA Isolation

From olive pericarp (fruit mesocarp and epicarp) tissues, total RNA was extracted following Gomez-Jimenez et al. (2010). The RNA purity and concentration were established by scanning UV spectroscopy. The resulting RNA was employed in the qRT-PCR analysis.

### Quantitative RT-PCR

Previously published RNA-Seq data (Gil-Amado and Gomez-Jimenez, 2013) were mined for sphingolipid-related genes. Expression levels were determined for genes encoding the serine palmitoyltransferase I (*OeSPT/LCB1*, Unigene ID: OL002160), sphingosine kinase (*OeSPHK*, Unigene ID: OL002715), alkaline dihydroceramidase (*OeACER*, Unigene ID: OL002183) and glucosylceramidase (*OeGlcCerase*, Unigene ID: OL002165). With random hexamers and Superscript III (Invitrogen), total RNA (2 mg) was reverse transcribed, following the manufacturer’s indications. For qRT–PCR assays, purified cDNA (2 ng) served as a template, using gene-specific primers. Primer sequences were 5′-GGTGTTGGTTCCTGTGGTCCTCGTGGA-3′ (forward) and 5′-AGACATAACCAGAGCTACTGAGGCGTT-3′ (reverse) for *OeSPT/LCB1*; 5′-GGCCCTTTTGTTTCAATCTGGCT T-3′ (forward) and 5′-ACCTCTGGCCAGAACCTCACCATC-3′ (reverse) for *OeSPHK*; 5′-ATGGAAGATGGATTATCAAGC-3′ (forward) and 5′-AGGAAGAATGACATAATTTGG-3′ (reverse) for *OeACER*, and 5′-GGTCAATGTAATGGACAGGAT-3′ (forward) and 5′-CGGAATTGTCAAAGCACTTTGC-3′ (reverse) for *OeGlcCerase*. A SYBRGreen-PCR Master kit (Applied Biosystems) containing an AmpliTaq Gold polymerase on an iCycler (BioRad Munich) was used for cDNA amplification following the supplier’s protocol. The samples were submitted to thermal-cycling conditions to activate DNA polymerase (94°C, 45 s at 55°C, 45 s at 72°C, and 45 s at 80°C). A final 7-min elongation step was performed at 72°C. The melting curve was programmed to rise 0.5°C every 10 s, starting at 62°C. The amplicon, submitted to electrophoretic analysis, was sequenced for identity confirmation. An estimation of qRT-PCR efficiency was made by a calibration dilution curve and slope calculation. The expression levels were established as the number of cycles that the amplification needed before reaching a threshold set for the exponential phase of the PCR (CT). The data were normalized based on the amount of the *O. europaea* ubiquitin (*OeUB*) gene (Gomez-Jimenez et al., 2010; Gil-Amado and Gomez-Jimenez, 2012). Duplicates were utilized from three biological replicates in two separate experiments.

## Results

### Fruit Developmental Stages and Lipid Content

Olive (cv. Picual) fruits completed their development and ripening in 210 days under the experimental conditions used. Five major developmental stages were established to better scrutinize olive-fruit development. **Figure 1** shows representative images of the olive fruit harvested at 70 (stage 1, completed seed/pit hardening), 125 (stage 2, pericarp development), 168 (stage 3, onset of ripening), 182 (stage 4, veraison), and 210 (stage 5, completed fruit ripening) DPA used for this work. In olive fruit, firmness decreased following the onset of ripening and reached the oversoft stage at 210 DPA (**Figure 1**). The content of total lipids (% DW) was determined throughout these stages in the fruit pericarp (mesocarp and epicarp). We found that the amount of the total lipid content progressively increased during fruit development (**Figure 1**). Small amounts of lipids accumulated during the first phase of development (70 DPA), but the lipid level maintained a continued increase throughout ripening until reaching 7.5-fold the departing lipid level in the fruit (**Figure 1**).

**FIGURE 2:**
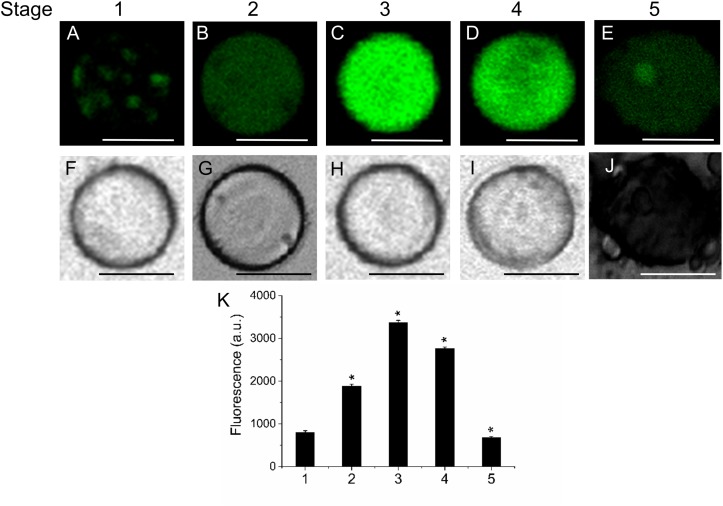
Lipid distribution in live protoplasts from olive fruit during development. **(A–E)** Bodipy stained protoplasts from the olive fruit at different developmental stages compared to the white-light image **(F–J)**. Olive fruit were sampled at 1 (green with completed pit hardening), 2 (green with peak of cell expansion), 3 (onset of ripening), 4 (veraison), and 5 (ripe) stages of development. **(K)** Quantification of Bodipy fluorescence in live protoplasts from olive fruit at different stages of development. Data from about 20 protoplasts were acquired per sample, and data were compiled using FV10 4.2 software (Olympus). Columns and bars indicate means ± SD, respectively, from five independent experiments. Statistically significant differences from the preceding point based on unpaired Student’s *t*-test at *P* < 0.05 are denoted by asterisk. Scale bars are 10 μm.

Next, we investigated the level of lipids during fruit growth and ripening through the use of several fluorescent probes that allow differentiating the type of lipid and its location. This was performed in live protoplasts, which improved plasma-membrane accessibility to the individual lipid analogs supplemented from outside the cell, and also helped to diminish non-specific fluorescence signals, ascribed mainly to the non-specific staining of cell-wall components. Neutral and polar lipid contents were located in live protoplasts from olive fruit during development by Bodipy and Nile Red staining, respectively. As shown in **Figure 2**, during olive-fruit development, the Bodipy fluorescence intensity gradually increased (**Figures 2A–C**), reaching a maximal at the onset of ripening (stage 3) (**Figure 2C**) and then, significantly decreased during the late stage of fruit development (stage 5, full ripe fruit) (**Figures 2D,E**). For white-light images, see **Figures 2F–J**. Intensity of the signal is shown in **Figure 2K**, wherein the biphasic pattern is appreciated.

**FIGURE 3:**
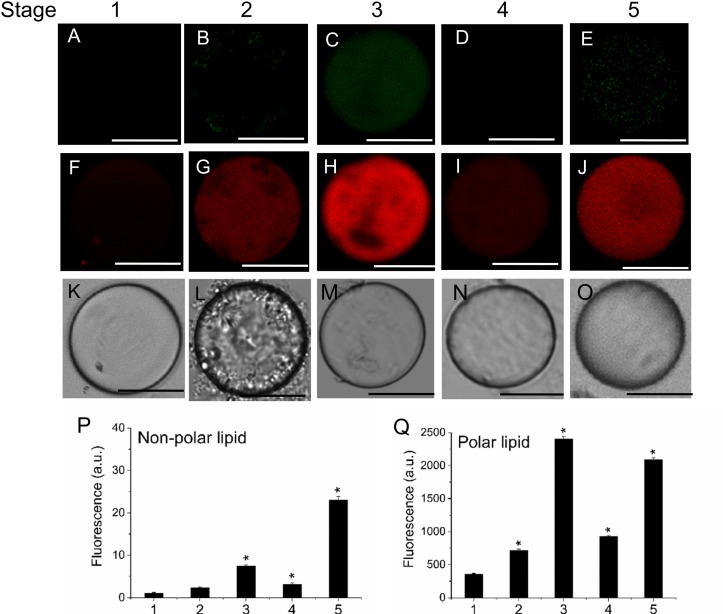
Distribution of non-polar and polar lipids of live protoplasts from olive fruit during development. Fluorescence of variable color from protoplasts indicated a difference in neutral lipid composition: neutral or non-polar lipid appeared as green **(A–E)** and polar lipid appeared as red **(F–J)**. **(A–J)** Nile Red stained protoplasts from the olive fruit at different stages of development compared to the white-light image **K–O**). Scale bars are 10 μm. **(P,Q)** Quantification of Nile Red fluorescence in live protoplasts from olive fruit at different stages of development. The orange/red fluorescence of Nile Red was acquired with a 630 nm long-pass filter, and the mean fluorescence intensities of olive-fruit protoplasts were measured in a logarithmic scale. Data from about 20 protoplasts were acquired per sample, and data were compiled using FV10 4.2 software (Olympus). Columns and bars indicate means ± SD, respectively, from five independent experiments. Statistically significant differences from the preceding point based on unpaired Student’s *t*-test at *P* < 0.05 are denoted by asterisk.

Likewise, the use of fluorescent Nile Red for lipid measurement in olive-fruit protoplast revealed that the onset of ripening (stage 3) induced a high increase in polar-lipid concentration, while an enrichment of non-polar lipids was slightly detected in the fully ripe fruit (stage 5) (**Figures 3E,P**). Polar lipids, which are present mostly in membranes, are stained in red (**Figures 3F–J**) whereas neutral lipids (esterified cholesterol and triglycerides), which are present in lipid droplets, are stained in green (**Figures 3A–E**). Therefore, the maximum concentrations of polar lipids were significantly correlated with the onset of fruit ripening in olive (**Figure 3Q**). **Figures 3K–O** depict white-light images from protoplasts.

### Visualization of Sphingolipid-Enriched Plasma-Membrane Regions and Vesicle Trafficking during Olive-Fruit Development and Ripening

**FIGURE 4:**
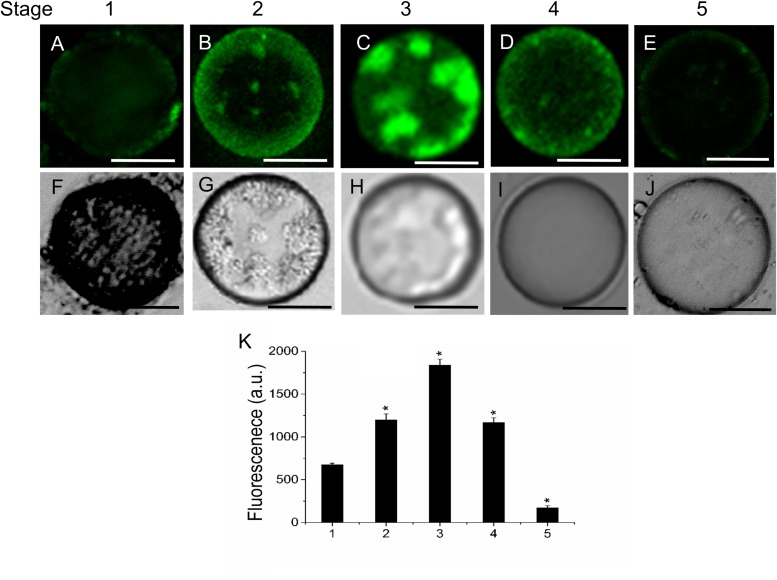
Changes in sphingolipid content in live protoplasts from olive fruit during development. **(A–E)** BD-SM stained protoplasts from the olive fruit at different stages of development compared to the white light image **(F–J)**. Scale bars are 10 μm. **(K)** Quantification of BD-SM fluorescence in live protoplasts from olive fruit at different stages of development. Data from about 20 protoplasts were acquired per sample, and data were compiled using FV10 4.2 software (Olympus). Columns and bars indicate means ± SD, respectively, from five independent experiments. Statistically significant differences from the preceding point based on unpaired Student’s *t*-test at *P* < 0.05 are denoted by asterisks.

To determine the distribution pattern of sphingolipids during fruit development and ripening, live olive protoplasts were stained with Bodipy Sphingomyelin FL C12 (BD-SM). As shown in **Figure 4**, BD-SM fluorescence increased in fruit protoplasts during pericarp-cell expansion and showed the highest peak of BD-SM accumulation at the stage 3 (**Figures 4A–E,K**). The signal was detectable inside the protoplast (intracellular membranes) and in plasma-membrane. At the veraison (stage 4), the fluorescence intensity gradually decreased (**Figures 4D,K**). These results suggest that sphingolipid domains formation is required during olive-pericarp cell expansion and at the onset of ripening, most probably to mediate translocation of cargo proteins to plasma-membrane involved in endo- or exocytosis.

**FIGURE 5:**
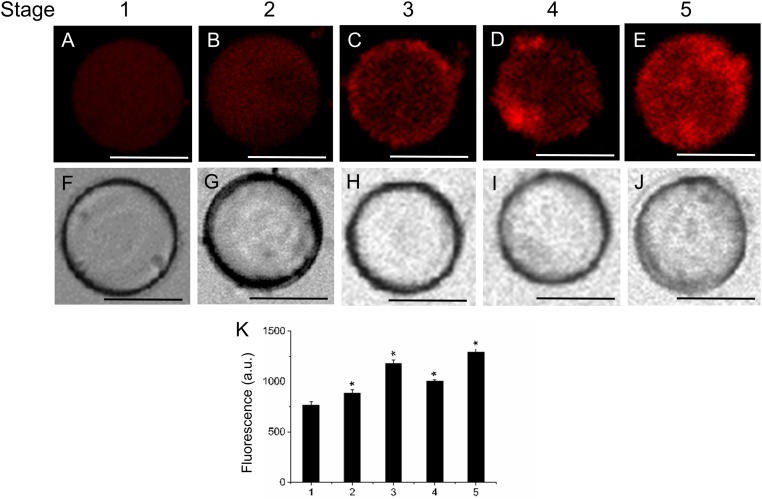
Changes in vesicle trafficking in live protoplasts from olive fruit during development. **(A–E)** FM4-64 stained protoplasts from the olive fruit at different stages of development compared to the white light image **(F–J)**. Scale bars are 10 μm. **(K)** Relative FM4-64 internalization fluorescence intensity in live protoplasts from olive fruit at different stages of development. Data from about 20 protoplasts were acquired per sample, and data were compiled using FV10 4.2 software (Olympus). Values are means ± SD (*n* = 20 protoplasts). Statistically significant differences from the preceding point based on unpaired Student’s *t*-test at *P* < 0.05 are denoted by asterisks.

Because sphingolipids have been reported to be involved in endomembrane trafficking (Markham et al., 2013), we analyzed the vesicle trafficking in fruit protoplasts during fruit ripening. We used FM4-64 to monitor endocytosis in live protoplasts during olive-fruit development. As shown in **Figure 5**, the FM4-64 dye was internalized and a substantial number of punctuated fluorescent vesicles were detected in the cytosol of olive protoplasts during fruit development. Conversely, few fluorescent vesicles were observed in fruit protoplasts at stage 1, indicating that endocytosis was stimulated in protoplast cell derived from olive fruit during cell expansion and ripening (**Figure 5**). Quantification of FM4-64 showed a significant increase of the probe in fruit protoplasts at the fully ripe stage compared with fruit protoplasts at the veraison stage (**Figure 5K**), indicating that endocytosis was stimulated during olive-fruit ripening.

### Sphingolipid LCB Content and Composition during Olive-Fruit Development and Ripening

**FIGURE 6:**
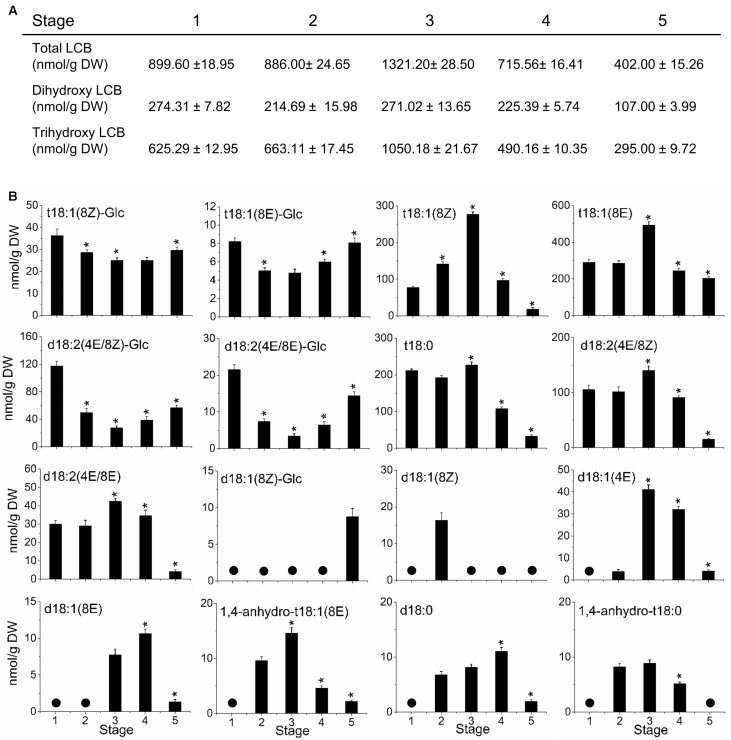
Sphingolipid content and composition of olive fruit during development. **(A)** Content of LCB hydrolyzed from total sphingolipids and relative content of dihydroxy or trihydroxy LCBs in total extracts. **(B)** Profiles of LCBs from olive fruit at different stages of development. Data are means of three independent biological repeats. Statistically significant differences from the preceding point based on unpaired Student’s *t*-test at *P* < 0.05 are denoted by asterisks. The molecular species not detected are denoted by a circle 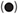. d18:1(8*E*)-Glc and 1,4-anhydro-t18:1(8*Z*) were undetectable in olive fruit at five different stages of development.

To investigate the possible relationship between sphingolipid composition and fruit development, we analyzed the LCB profiles in the olive fruit during development and ripening. The amount of LCB hydrolyzed from total sphingolipids changed during olive-fruit development (**Figure 6**). The total LCB content did not change significantly during the cell-expansion stage (stage 2), whereas it increased up at the onset of ripening (stage 3) and then, declined during the last phases of olive-fruit ripening (stage 4 and 5) (**Figure 6A**). The total LCB increase was due essentially to the increase of trihydroxy LCBs (**Figure 6A**). In fact, the fraction of trihydroxy LCBs was the main contributor to the total LCB levels at the five development stages. Therefore, the behavior of trihydroxy LCB content was similar to the trend observed for the total LCB, and the highest peak of accumulation was observed at the onset of fruit ripening (stage 3). However, dihydroxy LCB levels were not altered during olive-fruit development, except in the last phase of fruit ripening (stage 5, fully ripe fruit), when the lowest levels were measured (**Figure 6A**). Thus, at the onset of olive fruit ripening, the main LCB species showing an augment were trihydroxylated LCBs in their saturated and unsaturated forms (8*Z* and 8*E*) (**Figure 6B**). Altogether, these results indicate, first, that the trihydroxy LCBs potentially play a role at the onset of ripening, and second, that levels of sphingolipids must drop to reach fruit ripening.

In particular, at onset of ripening or stage 3, olive fruit showed the highest peak of LCB accumulation with a rise in t18:1(8*E*) > t18:1(8*Z*) > t18:0 > d18:2 (4*E*/8*Z*) > d18:2 (4*E*/8*E*) > d18:1(4*E*) > 1,4-anhydro-t18:1(8*E*) levels that coincided with a fall in t18:1(8Z)-Glc, t18:1(8E)-Glc, d18:2(4E/8Z)-Glc, and d18:2(4E/8E)-Glc levels (**Figure 6B**). Conversely, in the olive fruit at the fully ripe stage (stage 5), a rise was registered in the t18:1(8Z)-Glc, t18:1(8E)-Glc, d18:2(4E/8Z)-Glc, and d18:2(4E/8E)-Glc levels, while t18:1(8Z), t18:1(8E), t18:0, d18:2(4E/8Z), d18:2(4E/8E), d18:1(4E), and 1,4-anhydro-t18:1(8E) levels fell in comparison with the fruit at the onset of ripening (**Figure 6B**). Likewise, our data show an increase of d18:0 and d18:1(8*E*) content in olive fruit at the veraison stage (**Figure 6B**).

### Expression of Sphingolipid-Related Genes during Olive-Fruit Development

**FIGURE 7:**
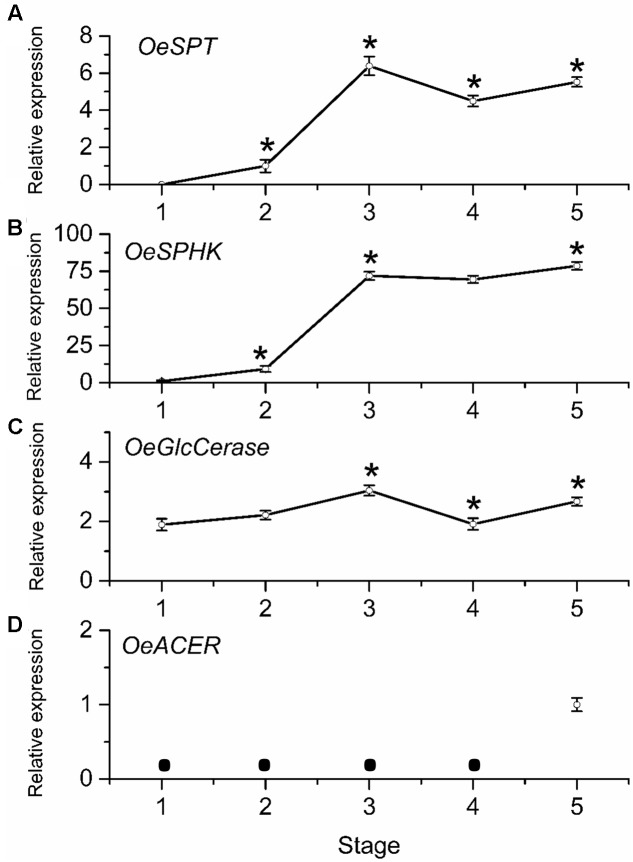
Gene expression of *OeSPT* **(A)**, *OeSPHK* **(B)**, *OeGlcCerase* **(C)**, and *OeACER* **(D)** mRNAs in olive fruit during development. Total RNAs were isolated from olive fruit at five different stages of development. Data are the means ± SD of three biological replicates with three technical repeats each and were obtained by qRT-PCR normalized against *O. europaea* ubiquitine. Statistically significant differences from the preceding point based on unpaired Student’s *t*-test at *P* < 0.05 are denoted by asterisks. The mRNA not detected is denoted by a circle 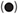.

To determine whether mRNA levels of sphingolipid-metabolism-related genes were differentially regulated during olive-fruit development and ripening, we assessed the expression profiles of *OeSPT*, *OeSPHK*, *OeGlcCerase*, and *OeACER* genes in olive fruit using real-time RT-PCR (**Figure 7**). The *OeSPT*, *OeSPHK*, and *OeGlcCerase* genes were differentially expressed throughout the course of olive-fruit development (**Figures 7A–C**), whereas *OeACER* gene expression was detected only at the fully ripe stage (**Figure 7D**), when the content of total LCBs (dihydroxy and trihydroxy forms) diminished (**Figure 6A**). The highest *OeSPT* and *OeGlcCerase* expression was found in olive fruit at the onset of ripening (stage 3), while the highest *OeSPHK* and *OeACER* expression was found in olive fruit at the end of fruit ripening (stage 5) (**Figure 7**).

From 70 (stage 1) to 168 (stage 3) DPA, *OeSPT*, *OeSPHK*, and *OeGlcCerase* expression increased in olive fruit (**Figures 7A–C**), suggesting that *OeSPT*, *OeSPHK*, and *OeGlcCerase* genes were up-regulated during pericarp cell expansion and at the onset of ripening. However, from 168 (stage 3) to 182 (stage 4) DPA, *OeSPT*, and *OeGlcCerase* expression decreased in olive fruit (**Figures 7A,C**) while *OeSPHK* gene expression did not significantly change (**Figure 7B**). Finally, in ripe fruit at 200 DPA (stage 5), *OeSPT*, *OeSPHK*, and *OeGlcCerase* transcript levels rose in comparison with those observed at 182 DPA (H) (**Figure 7**). Thus, our data indicate that the genes *OeSPT*, *OeSPHK*, and *OeGlcCerase* were positively associated with the pericarp cell expansion and the onset of ripening, while *OeACER* gene was up-regulated in olive fruit at the late stage of ripening. In particular, *OeSPT* and *OeGlcCerase* showed significantly higher expression in association with the onset of ripening and in parallel to the increase of LCB content.

## Discussion

Recent research has elucidated many specific aspects concerning the functions of sphingolipids in plants (Luttgeharm et al., 2016), although the part they play in fruit development or ripening (a plant-specific process) remains unclear.

**FIGURE 8:**
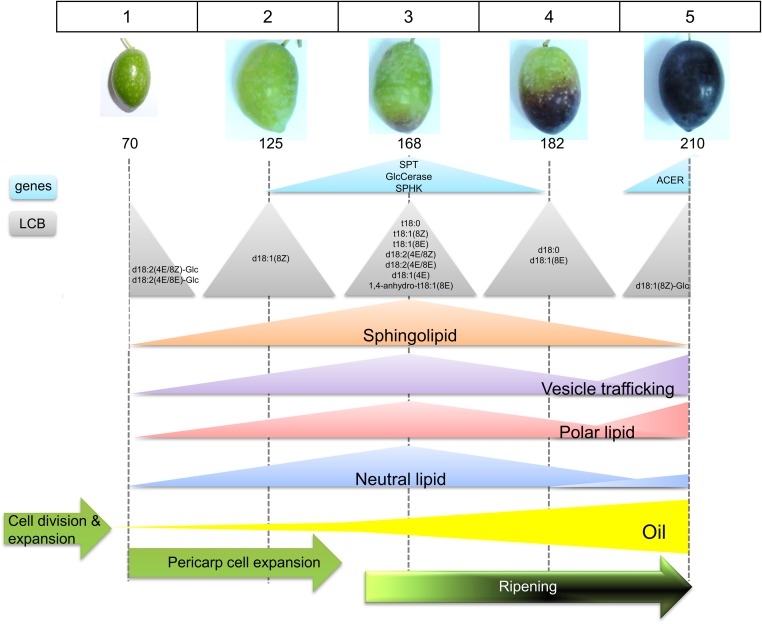
Scheme representing the sphingolipid profiling during the phases of olive fruit development. See text for details.

No reports are available on the sphingolipid content and gene expression analysis during fleshy-fruit development. Here, the neutral:polar lipid ratio and sphingolipid content are studied employing dye, fluorescence labeling, and confocal fluorescence microscopy image analysis, as well as the sphingolipid LCB composition and sphingolipid-related gene expression during fruit development and ripening in olive. It is shown that the measured proportion both of polar lipids and sphingolipids in live protoplasts from olive fruits as well as sphingolipid LCB composition and the expression of some genes related to sphingolipid metabolism display significant fluctuations during key points of the stages of olive-fruit development and ripening (**Figure 8**).

Here, the evaluation performed throughout the different developmental stages of fruit indicated an increase in sphingolipids rich in trihydroxylated LCBs in olive fruit at the onset of fruit ripening. This coincided with a larger expression of the *OeSPT/LCB1* gene from the biosynthetic SPT enzyme as well as the major signal detection from the probes for sphingolipid-enriched plasma-membrane regions and for the endocytosis activity. In addition, these parameters agreed with the probe monitoring the increase in polar lipids as well. Likewise, the data from our study indicate that endocytosis, as visualized after fluorescent-dye FM4-64 staining, underwent strong stimulation during the onset of ripening and was prolonged until stage 5. In contrast, at the veraison stage, the amount of trihydroxy-sphingolipids and polar lipids decreased by more than a half. This result was consistent with a down-regulation of *SPT* and *OeGlcCerase* expression at the same stage. The metabolism during the veraison process is very active, including lipid accumulation, alterations in metabolites, softening, and coloring (Conde et al., 2008). Specifically, the softening results from changes in cell-wall structure caused by the action of cell-wall-modifying enzymes (Conde et al., 2008; Karlova et al., 2014). This would be favored by the specialized sphingolipid-enriched membrane regions involved in cell-wall modification.

Olive-fruit development and growth implies reserve deposition together with cell multiplication and expansion. The results shown here indicate that *de novo* lipid synthesis is required in order to sustain the large accumulation of total lipids that olive-fruit ripening demands. While triglycerides constitute the main reserve lipid (Chapman and Ohlrogge, 2012; Hernández et al., 2016), the vast proportion of *de novo* synthesized sphingolipids contribute to fulfill the structural part of the membrane matrix, since sphingolipids such as LCBs or ceramides, which display a signaling function, are usually found in very small amounts (Saucedo-García et al., 2011). The relationship between trihydroxy sphingolipids with the growth of Arabidopsis has been reported. Double mutants (*sbh1 sbh2*) of *Sphingoid Base Hydroxylase* (*SBH*) genes, exhibiting a complete loss of trihydroxy LCBs, presented reduced growth caused by defects in cell elongation and cell division (Chen et al., 2008). In accordance, overexpression of both genes produced larger plants. Additionally, the *cer10* mutant, affected in enoyl-CoA reductase activity involved in VLCFA biosynthesis, showed lower relative amounts of VLCFA, a smaller size, and morphological abnormalities in all the aerial organs. This phenotype was correlated with the reduction in cell size and aberrant cell expansion (Zheng et al., 2005). Thus, we suggest that trihydroxy-LCB sphingolipids containing VLCFAs are crucial for olive-fruit growth. Cell multiplication and cell enlargement required for fruit growth demand high amounts of membrane lipids in order to increase the bilayer surface area, mainly for the plasma and vacuole membranes. Sphingolipids are abundant lipids in plant cells. About 40% of the lipids from Arabidopsis plasma-membranes are complex sphingolipids and trihydroxylated LCBs is the most represented fraction. Likewise, the identification of plasma-membrane regions enriched in sphingolipids were associated with the onset of ripening, indicating that they may be involved in endocytic activities necessary for cell-wall remodeling during olive-fruit ripening.

At the completed fruit ripening stage, the lowest levels of either dihydroxy- or trihydroxy-sphingolipids and the highest accumulation of non-polar lipids was detected in olive fruit. These results suggest that when VLCFAs are not part of trihydroxy sphingolipids they could be acting as components of triacylglycerols (TAGs) and accumulate in the fleshly pericarp. A connection of sphingolipid and glycerolipid metabolism through the fatty acid flux has previously been suggested (Chen et al., 2012). This is feasible in our case, given that in the olive fruit the TAGs localized in the pulp are not used to feed the olive embryo (Salas et al., 2000). The oil from olive has the major content of TAGs with VLCFA in comparison to avocado and palm oils (Salas et al., 2000), supporting our hypothesis. In addition, VLCFAs could repress cell proliferation by suppressing cytokinin biosynthesis at the end of ripening, as happens in the vasculature of Arabidopsis (Nobusawa et al., 2013), given that the duration of cell proliferation is the main determinant of organ size and shape.

Because changes in sphingolipid metabolism during fruit development and ripening may be part of a mechanism controlling the transitions from pericarp-cell expansion to ripening, sphingolipid levels should be maintained within strict limits. The onset of ripening, as mentioned before, is associated with strong increase in total LCB content in olive fruit. However, the profiling of sphingolipid LCB measurement revealed a specific association between the individual LCBs and the stages of fruit development and ripening. The sphingolipids containing d18:1(8*Z*) were found specifically in olive fruit at the stage 2 (cell expansion) and those with d18:1(8*Z*)-Glc appeared specifically in olive fruit at the stage 5 (fully ripe fruit), suggesting that these sphingolipids may play a specific role during olive-fruit development and ripening. This difference is clear when the relative distribution of the LCBs is compared during olive-fruit ripening. At the onset of ripening, an increase in the t18:1(8*E*), t18:1(8*Z*), t18:0, d18:2 (4*E*/8*Z*), d18:2 (4*E*/8*E*), d18:1(4*E*), and 1,4-anhydro-t18:1(8*E*) levels, and increased *OeSPT*, *OeSPHK*, and *OeGlcCerase* expression parallels a decline of the t18:1(8*Z*)-Glc, t18:1(8*E*)-Glc, d18:2(4*E*/8*Z*)-Glc, and d18:2(4*E*/8*E*)-Glc levels. Conversely, the olive-fruit ripening shows, on the one hand, an increase in the t18:1(8*Z*)-Glc, t18:1(8*E*)-Glc, d18:2(4*E*/8*Z*)-Glc, and d18:2(4*E*/8*E*)-Glc levels and, on the other hand, a significant decrease in the t18:1(8*Z*), t18:1(8*E*), t18:0, d18:2(4*E*/8*Z*), d18:2(4*E*/8*E*), d18:1(4*E*), and 1,4-anhydro-t18:1(8*E*) levels. The data indicate that these LCB species maintain an inverse correlation during olive-fruit ripening, suggesting an antagonistic action of these LCB species during olive-fruit ripening.

Currently, little is known concerning sphingolipid turnover rates, or about the way in which sphingolipid catabolism contributes to membrane function or overall plant responses to changing conditions in the environment (Luttgeharm et al., 2016). Here, the identification of four genes of the sphingolipid biosynthesis and turnover pathways, expressed during olive-fruit development and ripening, has been described. Because of the multiple functions of sphingolipids in plants, none of the four olive-fruit sphingolipid-related genes was fruit specific (Gil-Amado and Gomez-Jimenez, 2013). However, *OeACER* expression has been found exclusively in fully ripe olive fruit and showed a specific association with the d18:1(8*Z*)-Glc accumulation. On the basis of mutant phenotypes, in Arabidopsis, the enzyme AtACER was found to influence turgor pressure within pollen tubes as well as guard cells of siliques (Chen et al., 2015); however, the function of ACER in fleshy-fruit ripening remains to be determined. Our results suggest that *OeACER* is a ripening-inducible gene and are related to the last stage of olive-fruit ripening. On the other hand, our data demonstrate a correlation between the *OeSPT* and *OeGlcCerase* gene expression and LCB levels during olive-fruit development, implying that the synthesis of 3-ketosphinganine and the hydrolysis of glucosylceramide into glucose and ceramide might be a rate-limiting step in the biosynthesis and turnover of sphingolipids, respectively. A possible participation of the ORM proteins, SPT negative regulators must be considered (Kimberlin et al., 2016). These results support the hypothesis that SPT and GlcCerase could be involved in olive-pericarp cell expansion as well as in fruit ripening.

## Conclusion

Molecular and microscopic analyses reveal that sphingolipid metabolism dynamically participates during olive fruit ripening. There is a differential content of sphingolipid species, as well as preferential membrane distribution and gene expression throughout the ripening process. This is the first report available describing the regulation of sphingolipid distribution, content and gene expression during fleshy-fruit development and ripening, a plant-specific process. This detailed temporal profile of the LCB composition and the sphingolipid gene expression in the olive fruit offers new insights for understanding the physiological role(s) of plant sphingolipids.

## Author Contributions

CI and MP-L performed the experiments. MP and JL sampled the material and contributed to sphingolipid analysis. MG contributed to data analysis. MS-G and MG-R contributed to analyze and discuss the results and prepare the manuscript. MG-J conceived and supervised the study, wrote and critically revised the manuscript.

## Conflict of Interest Statement

The authors declare that the research was conducted in the absence of any commercial or financial relationships that could be construed as a potential conflict of interest.

## Supplementary Material

FIGURE S1 Viability was successfully tested using trypan blue, showing a protoplast after trypan blue treatment; trypan blue is excluded from the cytosol of intact cells (here shown after BD-SM treatment). Scale bars are 5 μm.
