# Supplementary figures and images for: Sphingolipid Distribution, Content and Gene Expression during Olive-Fruit Development and Ripening
Source: Front Plant Sci. 2018 Jan 26;9:28. doi: 10.3389/fpls.2018.00028 (PMC5790798; doi:10.3389/fpls.2018.00028)

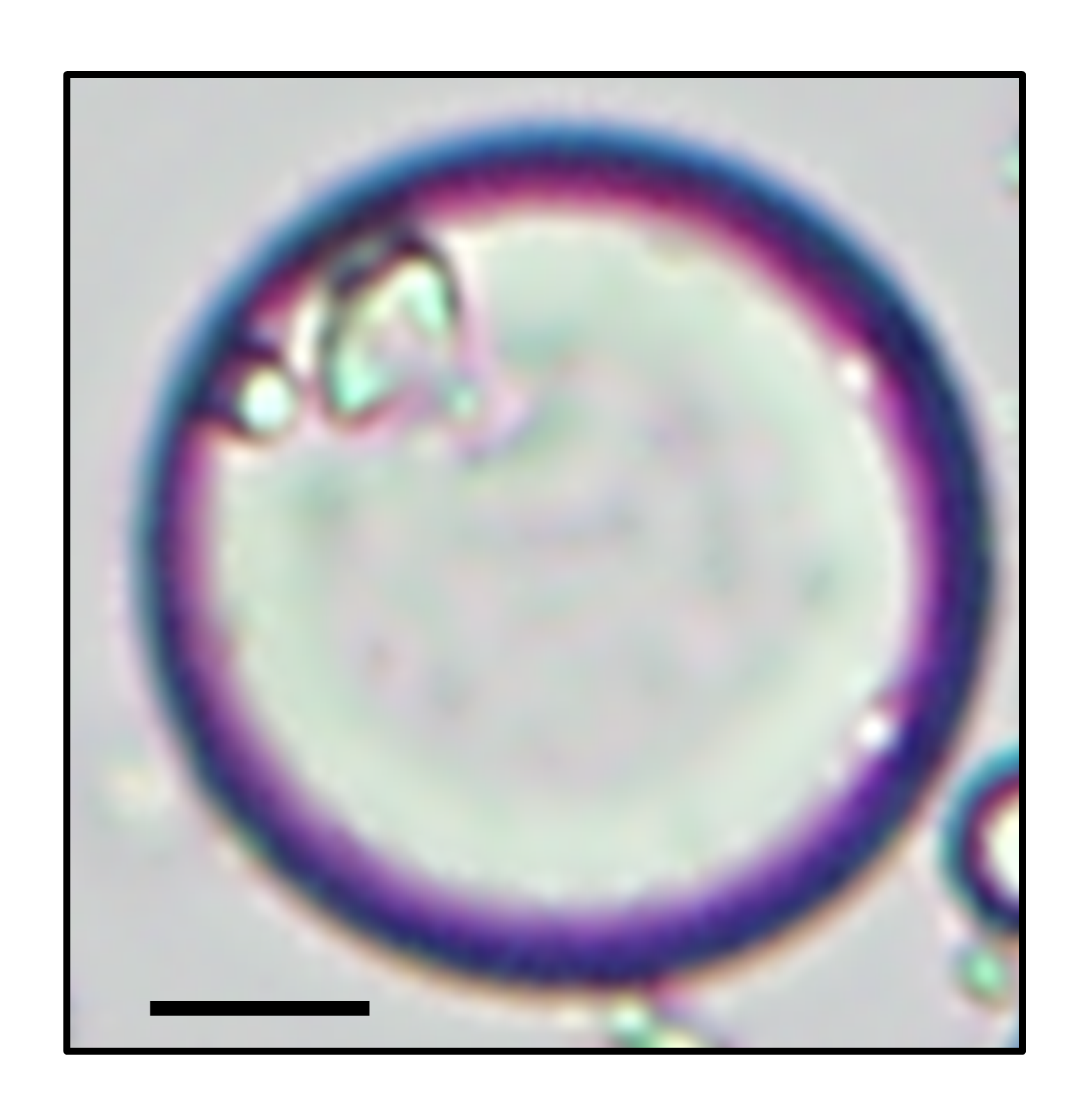

Supplement: FIGURE S1 — Viability was successfully tested using trypan blue, showing a protoplast after trypan blue treatment; trypan blue is excluded from the cytosol of intact cells (here shown after BD-SM treatment). Scale bars are 5 μm. [file Image_1.tif]
